# Preventive obesity agent montmorillonite adsorbs dietary lipids and enhances lipid excretion from the digestive tract

**DOI:** 10.1038/srep19659

**Published:** 2016-02-19

**Authors:** Pengfei Xu, Shu Dai, Jing Wang, Jun Zhang, Jin Liu, Fang Wang, Yonggong Zhai

**Affiliations:** 1 Beijing Key Laboratory of Gene Resource and Molecular Development, College of Life Sciences, Beijing Normal University, Beijing 100875, China; 2 Key Laboratory for Cell Proliferation and Regulation Biology of State Education Ministry, College of Life Sciences, Beijing Normal University, Beijing 100875, China; 3 Department of Medical Genetics and Cell Biology, School of Basic Sciences, Guangzhou Medical University, Guangzhou 511436, China; 4 Department of Biology Science and Technology, Baotou Teacher’s College, Baotou 014030, China

## Abstract

Western diets are typically high in fat and are associated with long-term complications such as obesity and hepatic steatosis. Because of the enjoyable taste of high-fat diets (HFDs), we are interested in determining how to decrease lipid absorption and enhance lipid excretion from the digestive tract after the consumption of eating fatty foods. Montmorillonite was initially characterized as a gastrointestinal mucosal barrier protective agent for the treatment of diarrhoea. Dietary lipid adsorbent- montmorillonite (DLA-M) was isolated and purified from Xinjiang montmorillonite clay *via* the water extraction method. Here, we show that DLA-M has an unexpected role in preventing obesity, hyperlipidaemia and hepatic steatosis in HFD-fed rats. Interestingly, combined application of polarized light microscopy and lipid staining analyses, showed that DLA-M crystals have dietary lipid-adsorbing ability *in vitro* and *in vivo*, which enhances lipid excretion *via* bowel movements. In summary, our results indicate that DLA-M prevent HFD-induced obesity. This novel dietary lipid-adsorbing agent can help prevent obesity and its comorbidities.

The prevalence of overweight and obesity is increasing worldwide, which raises concerns regarding the influence of these conditions on health. Overweight and obesity caused an estimated 3.4 million deaths worldwide in 2010^1^. A high body-mass index (BMI) is well established as a risk factor for various chronic diseases^2^, such as cardiovascular diseases, diabetes, cancer and musculoskeletal disorders, and for mortality^3^,^4^,^5^,^6^. Recent statistical analyses conducted on a global scale have revealed that 34.4% (33.2–35.5%) of adults are overweight (BMI ≥ 25), including 12% (11.5–12.5%) of adults who are classified as obese (BMI ≥ 30 kg/m^2^)^2^. Surveys conducted in the United States have predicted that the observed substantial increase in the prevalence of obesity may decrease future life expectancy^7^. Excess body weight severely affects quality of life. Furthermore, obesity has become a major global health challenge. Thus, identifying methods to prevent adiposity has become urgently needed in recent years.

Fatty foods are common in the daily diet, and such foods are so delicious that some people, particularly children, cannot resist eating them in excess. It is well established that fats greatly contribute to obesity. Thus, immobilizing consumed lipids and increasing lipid excretion to reduce lipid absorption in the digestive tract is important for preventing obesity. DLA-M, also known as montmorillonite, is a type of natural adsorbent clay mineral purified from bentonite, that is used as a major gastrointestinal mucosal barrier protective agent for the treatment of acute and chronic diarrhoea^8^. DLA-M is a layered aluminosilicate with adsorptive properties on either its external surfaces or within its interlaminar space^9^. Montmorillonite can adsorb organic substances, bacteria, viruses, heavy metal ions and other materials^10^,^11^, and it is widely used in industry, agriculture and medical treatment. However, no study has examined its ability to adsorb lipids; thus, it is unclear whether montmorillonite can prevent obesity by adsorbing lipids.

In the present study, we investigated the effects of DLA-M on increasing faecal lipid excretion *via* the immobilization of lipids in the gastrointestinal system. The results of *in vivo* HFD-fed animal experiments revealed that DLA-M affects faecal lipid excretion, thereby preventing adiposity under conditions of triglyceride (TG) and cholesterol overload. Taken together, these results demonstrate the potential for using DLA-M in modern health care and complementary and alternative medicine to prevent obesity.

## Results

### DLA-M prevented weight gain in HFD-fed rats

**Figure 1:**
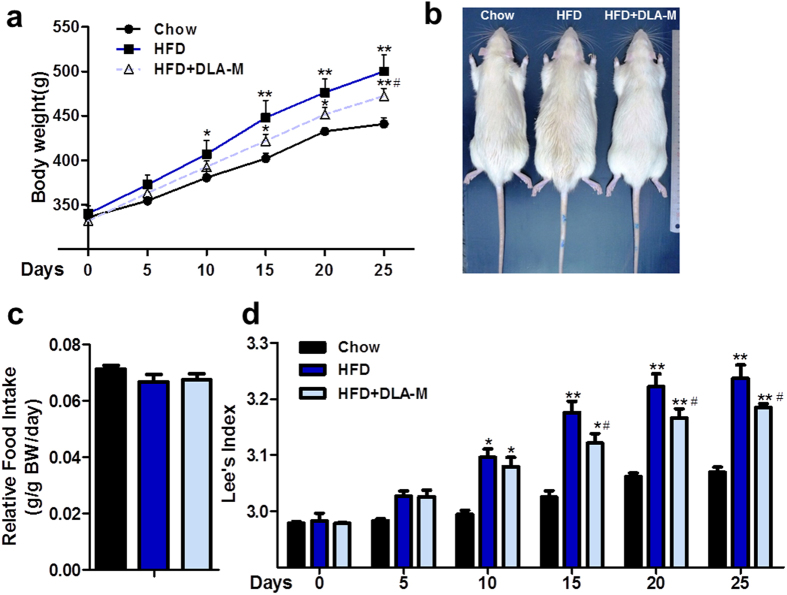
Effects of DLA-M on body weight gain in HFD-fed rats. Body weight (**a**), representative rats (**b**), relative food intake (**c**) and the Lee obesity index (**d**) in male rats fed a normal diet (Chow), high-fat diet (HFD) or HFD with DLA-M gavage (1 g/kg/day) for 25 days. Results are presented as the mean ± s.e.m. (n = 10 for each group). *P < 0.05; **P < 0.01 compared with Chow, ^#^*P* < 0.05 compared with HFD by one-way ANOVA with Tukey’s multiple comparison test.

To determine the effect of DLA-M on body weight gain, 2-month-old male Sprague Dawley (SD) rats were fed either a normal diet or a HFD for 25 days and were simultaneously treated with DLA-M (1 g/kg/day). HFD rats were significantly heavier than chow-fed rats after 5 to 10 days of feeding. This trend remained throughout the course of the treatment period. At the end of the 25-day treatment period, the rats in the HFD + DLA-M group weighed significantly less than the rats in HFD (Fig. 1a,b). Daily relative food intake did not differ between groups (Fig. 1c). The Lee obesity index reliably predicts the percentage of body fat in rats^12^. We calculated the Lee obesity index (weight ^0.33^/naso-anal length) for all rats (Fig. 1d).

### DLA-M inhibited adipocyte hypertrophy *in vivo*

**Figure 2:**
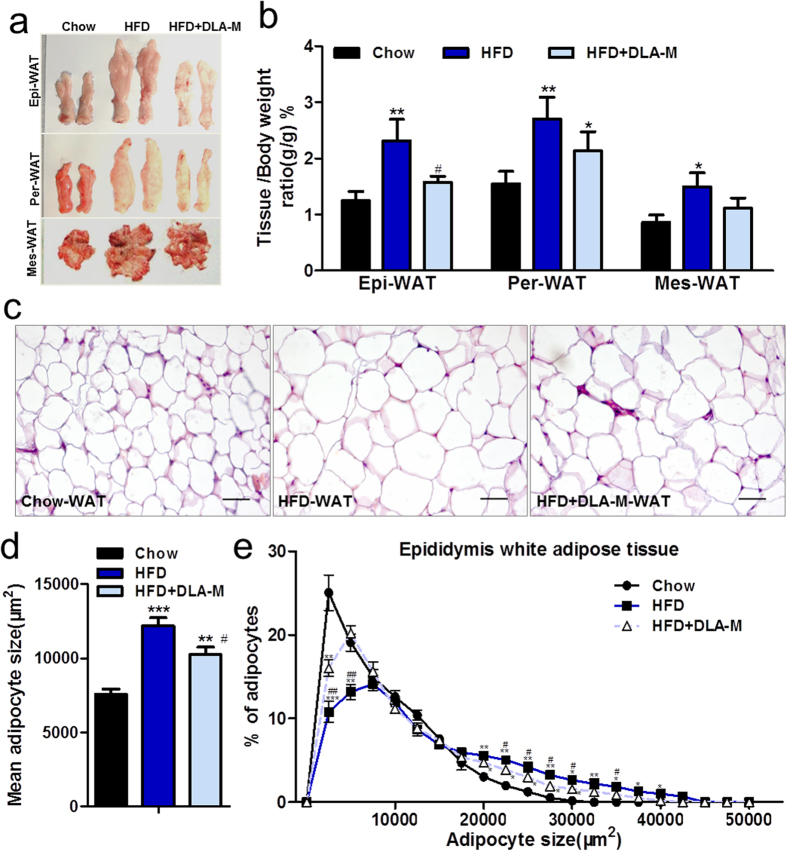
Treatment with DLA-M inhibited adipocyte hypertrophy *in vivo*. Gross appearance differences (**a**) and adipose tissue/body weight ratio (**b**) of rats (n = 10). (**c**) H&E staining of Epi-WAT sections from rats described in (**a**) (scale: 100 μm). Mean adipocyte size (**d**) and adipocyte size distribution (**e**) in each group (n = 5, 20 microscopic images were obtained per treatment group were). Results are shown as the mean ± s.e.m. *P < 0.05; **P < 0.01; ***P < 0.001, compared with Chow. ^#^*P* < 0.05; ^##^*P* < 0.01, compared with HFD by one-way ANOVA with Tukey’s multiple comparison test. Epi-WAT: epididymal white adipose tissue; Per-WAT: perirenal white adipose tissue; Mes-WAT: mesenteric white adipose tissue.

To determine whether DLA-M prevented weight gain by reducing the white adipose tissue content in rats, we isolated the three main types of white adipose tissues from the rats: epididymal white adipose tissue (Epi-WAT), perirenal white adipose tissue (Per-WAT) and mesenteric white adipose tissue (Mes-WAT). As shown in Fig. 2a, the amounts of all three tissue types were significantly increased by HFD feeding and are decreased by DLA-M treatment. The adipose tissue weights were normalized to the body weights; the DLA-M treatment significantly decreased the Epi-WAT/BW ratio (*P* < 0.05) compared with that in the untreated HFD rats (Fig. 2b). Although the Per-WAT/BW (*P* = 0.11) and Mes-WAT/BW (*P* = 0.07) ratios, were lower in the HFD + DLA-M-treated rats than in the HFD-fed rats, the differences were not statistically significant (Fig. 2b). Next, we examined the size of fat cells obtained from Epi-WAT sections (Fig. 2c). Adipocyte size was determined using automated imaging analysis. The HFD-fed and HFD + DLA-M-fed groups exhibited increased mean adipocyte size compared with the chow-fed group (+61.19%, *P* < 0.001 and +35.62%, *P* < 0.01, respectively). However, compared with the HFD-fed rats, the HFD + DLA-M-treated animals showed a mildly decreased adipocyte size (−15.87%, *P* < 0.05) (Fig. 2d). Analysis of the adipocyte size distribution revealed that DLA-M-treatment specifically increased the number of small adipocytes (2000–6000 μm^2^ in size) (Fig. 2e). Taken together, these results suggest that DLA-M prevented the obesity effect of the HFD.

### DLA-M decreased the hypercholesterolaemia induced by consumption of a HFD

**Figure 3:**
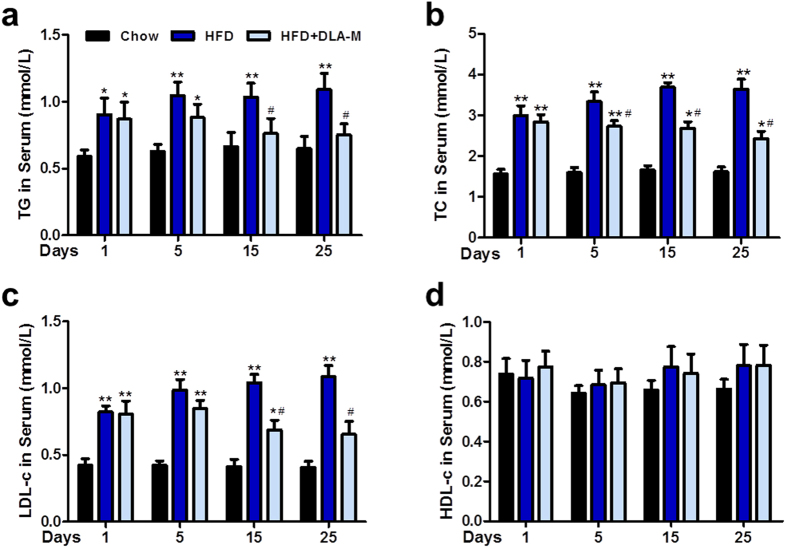
Serum lipid content analysis. Serum concentrations of TC (**a**), TG (**b**), LDL-c (**c**), and HDL-c (**d**) in rats fed a normal diet, HFD or HFD with DLA-M gavage for 25 days (n = 10). Results are shown as the mean ± s.e.m. *P < 0.05; **P < 0.01 compared with Chow, ^#^*P* < 0.05 compared with HFD by one-way ANOVA with Tukey’s multiple comparison test. TC: total-cholesterol; TG: triglyceride; LDL-c: low-density lipoprotein cholesterol and HDL-c: high-density lipoprotein cholesterol.

To examine the ability of DLA-M to decrease hyperlipidaemia, we measured the serum lipid concentration over time. To determine the duration of DLA-M treatment necessary for an effective reduction in serum lipid concentrations, we measured the serum lipid concentrations at specific time points. As shown in Fig. 3a–c, the serum lipid concentrations (TG, TC and LDL-c) were lower in HFD + DLA-M-treated group than in the HFD-fed group on the 15^th^ and 25^th^-days of treatment. At the end of the experiment, the TG, TC and LDL-c concentrations in the rats treated with DLA-M had decreased to 0.75 ± 0.08, 2.44 ± 0.17 and 0.65 ± 0.10 mmol/L, respectively (*P* < 0.05). No significant difference in HDL-c was observed between two groups (*P* > 0.05) (Fig. 3d). The decrease in serum lipid concentrations caused by DLA-M suggests that it decrease the extent of HFD-induced hypercholesterolaemia.

### DLA-M attenuated hepatic steatosis in the rat liver

**Figure 4:**
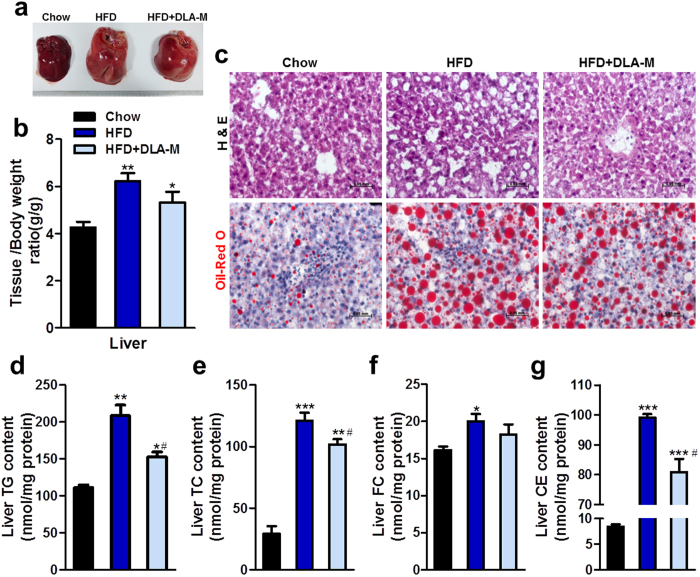
Effect of sustained DLA-M treatment on hepatic steatosis. Liver gross appearance (**a**) and liver/body weight ratio (**b**) of experimental rats (n = 10). H&E and Oil-Red-O staining of liver sections (**c**) from rats described in (**a**) (scale: 50 μm, liver lipids appear red). Hepatic TG (**d**), TC (**e**), FC (**f**) and CE (**g**) concentrations were analysed at the end of the study (n = 7). Results are shown as the mean ± s.e.m. *P < 0.05; **P < 0.01; ***P < 0.001 compared with Chow, ^#^P < 0.05 compared with HFD by one-way ANOVA with Tukey’s multiple comparison test. TG: triglyceride; TC: total cholesterol; FC: free cholesterol; and CE: cholesteryl ester.

Inhibition of whole body lipogenesis by DLA-M was hypothesized to meliorate hepatic steatosis. Gross observation indicated that the livers of the HFD-fed rats appeared hypertrophic due to fat accumulation, whereas the livers of the DLA-M-treated rats were nearly normal in size (Fig. 4a). As shown in Fig. 4b, DLA-M treatment slightly reduced the increase in the liver/BW ratio induced by HFD consumption, although this difference was not statistically significant (*P* = 0.07). To confirm whether DLA-M effectively attenuates liver steatosis, pathological examination of frozen liver sections was performed, and the results are presented in Fig. 4c. Haematoxylin and eosin (H&E) and Oil-Red O staining indicated that DLA-M effectively inhibited the liver fat infiltration and reduced the formation of lipid droplets. Subsequently, we analysed the liver lipid content normalized to the protein concentration. As expected, the liver lipid profile exhibited a similar pattern to the serum lipid profile. As shown in Fig. 4d–g, the HFD promoted the accumulation of lipids in the liver, and DLA-M treatment reduced the liver lipid levels. The levels of all lipids measured in the liver (TG, TC and CE) were lower in the HFD + DLA-M rats than in the HFD rats (−16.02%, −26.48% and −18.41%, respectively, *P* < 0.05). Taken together, these results show that dietary DLA-M reduces hepatic lipid accumulation and prevents the occurrence of hepatic steatosis in HFD-fed rats.

### DLA-M enhanced lipid excretion *via* bowel movements

**Figure 5:**
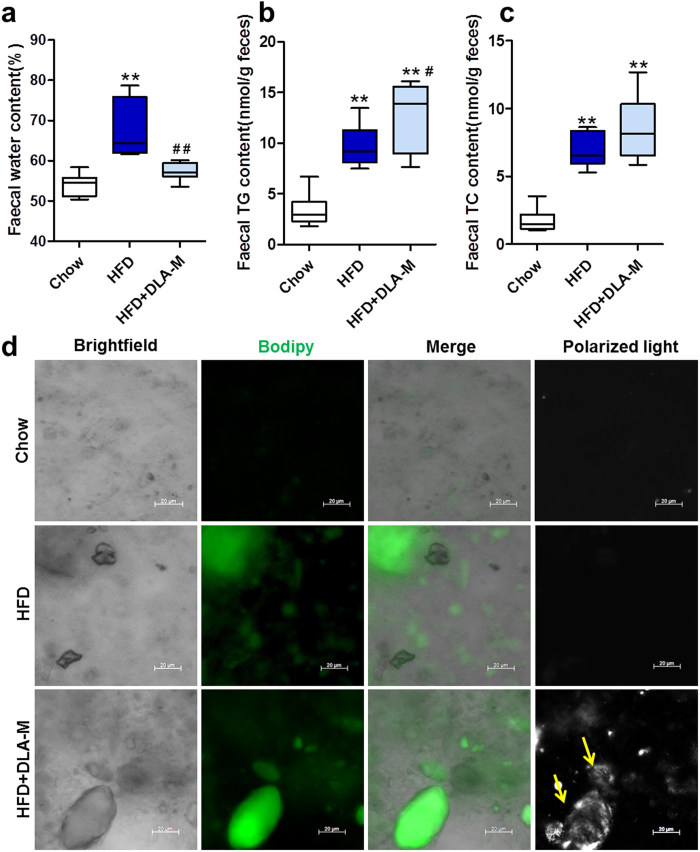
Effects of DLA-M on faecal lipid excretions. Box-plots of fecal faecal water (**a**), triglyceride (**b**) and total cholesterol (**c**) concentrations in the rats of each group (n = 8). (**d**) Bodipy staining of faecal smears (neutral lipids appear green) and polarized light microscopy examination of DLA-M crystals (yellow arrows) from rats of the indicated groups (scale: 20 μm). Results are shown as the mean ± s.e.m. **P < 0.01 compared with Chow, ^#^*P* < 0.05; ^##^*P* < 0.01 compared with HFD by one-way ANOVA with Tukey’s multiple comparison test.

The faecal water content was significantly higher in the HFD-fed rats than in the chow-fed rats (67.30 ± 2.74% vs 53.84 ± 1.23%, *P* < 0.01). Gavage treatment with DLA-M restored the faecal water content to near normal levels (57.36 ± 0.95%, *P* < 0.01) (Fig. 5a). The faecal TG and TC contents were higher in the HFD + DLA-M-fed rats, with the greatest difference observed for the TG content (Fig. 5b, *P* < 0.05). In addition, there were modest but in significant difference in fecal TC content between the HFD + DLA-M-treated rats and the chow-fed and HFD-fed rats (Fig. 5c, *P* = 0.06). Thus, we hypothesized that the increase in faecal lipid levels in the DLA-M-treated rats may have been due to the surface adsorption capability of DLA-M, which inhibited lipid absorption into the circulatory system. Faecal smears were stained with Bodipy (Fig. 5d). Polarized light microscopy revealed DLA-M crystals (yellow arrows).

### DLA-M immobilized dietary lipids *in vitro* and *in vivo*

**Figure 6:**
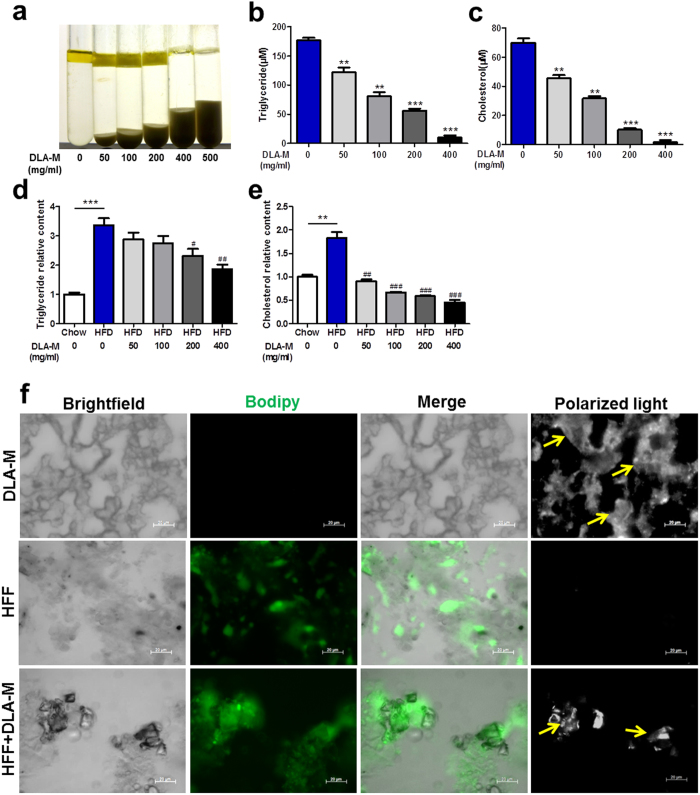
DLA-M fixed dietary lipids *in vitro*. (**a**), DLA-M adsorbs vegetable oils in glass tubes; treatment with 0–500 mg/ml DLA-M. 3T3-L1 mature adipocyte-conditioned medium treated with 0–400 mg/ml DLA-M: triglyceride (**b**) and cholesterol (**c**) content in the supernatant. Separated intestinal contents of mice fed a normal diet or HFD: saline was added (1:20), the samples were treated with 0–400 mg/ml DLA-M for 6 h, and then the relative contents of triglyceride (**d**) and cholesterol (**e**) were measured. (**f**), Bodipy staining of DLA-M, HFF and HFF + DLA-M smears (neutral lipids appear green) and polarized light microscopy examination of DLA-M crystals (yellow arrows), scale: 20 μm. The images were obtained from three replicate experiments. DLA-M, high-fat feed (HFF), and HFF + DLA-M (0.5 g of HFF and 200 μl of DLA-M (400 mg/ml) were added to the appropriate amount of distilled water to obtain smears. Results are shown as the mean ± s.e.m. **P < 0.01; ***P < 0.001compared with control, ^#^P < 0.05; ^##^P < 0.01; ^###^P < 0.001 compared with HFD by one-way ANOVA with Tukey’s multiple comparison test.

We further investigated how DLA-M immobilizes lipids. DLA-M adsorbs vegetable oils in glass tubes, as shown in Fig. 6a. Notably, the adsorption of oils by DLA-M was altered in a dose-dependent manner. Two other aluminosilicate clays, maifanite and laumonite, and the anion exchange resin colestyramine have no obvious oil adsorption abilities (Supplementary Fig. 1a, b). We treated 3T3-L1 mature adipocyte-conditioned medium, which contains large amounts of various lipids, with various concentrations of DLA-M and then measured the TG and cholesterol contents in the supernatant. As shown in Fig. 6b,c, DLA-M adsorbed TGs and cholesterol in a concentration-dependent manner.

Bile acids play a number of roles in regulating lipid metabolism^13^. To mimic the physiological conditions of the intestinal environment when bile is present, we isolated the intestinal chyme in mice fed a normal diet or HFD, added the appropriate amount of saline, treated the intestinal chyme and saline mixture with DLA-M, and then measured the relative contents of TGs and cholesterol. As shown in Fig. 6d,e, DLA-M adsorbed TGs and cholesterol in this system; however, it had nearly no ability adsorb bile acid (Supplementary Fig.2a, b).

**Figure 7:**
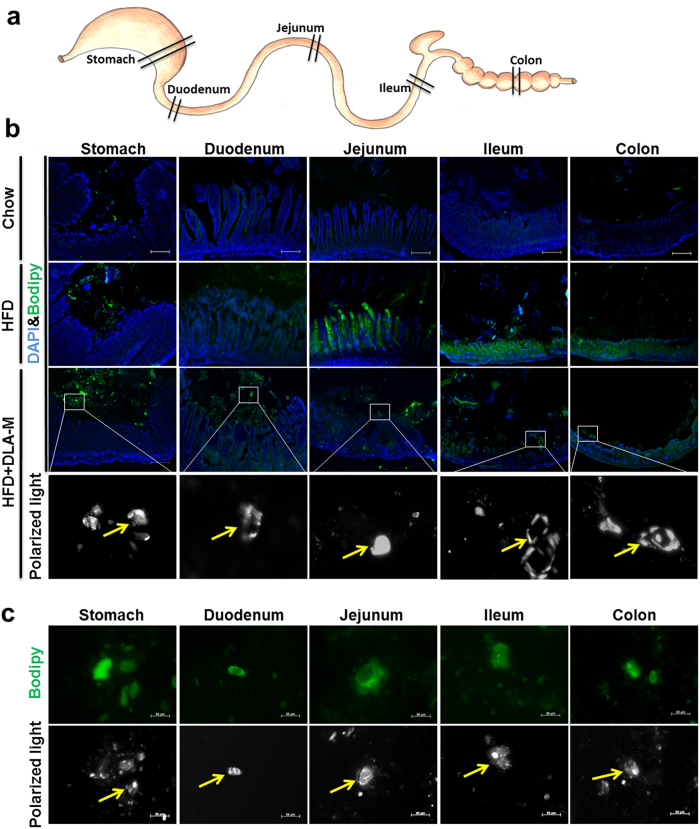
DLA-M immobilized lipids in the gastrointestinal system *in vivo*. (**a**) Schematic presentation of the sampling locations of the mouse digestive tract. (**b**) Bodipy & DAPI staining of digestive tract slices as described in (**a**) from mice fed a normal diet, HFD or HFD with DLA-M gavage (1 g/kg/day) for 3 days (scale: 200 μm). (**c**) Bodipy staining of gastrointestinal content smears of HFD + DLA-M mice; in the upper panels, neutral lipid appear green, and the lower panels polarized light microscopy examination images of DLA-M crystals (yellow arrows)(scale: 50 μm) (Chow, n = 3; HFD, n = 5; HFD + DLA-M, n = 5).

**Figure 8:**
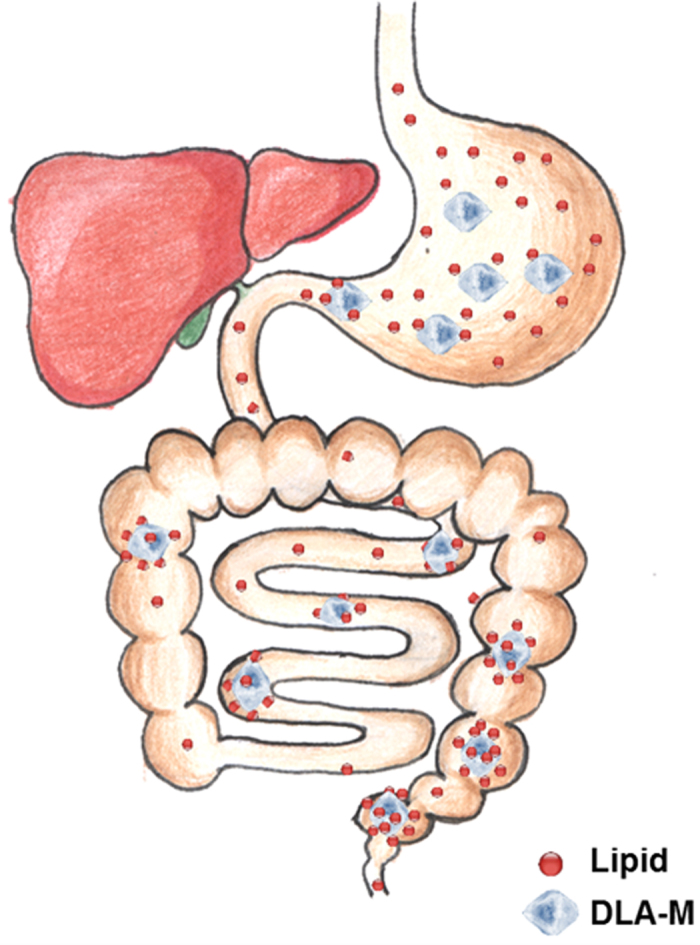
Model for DLA-M prevention of obesity development *via* fixation of dietary lipids and promotion of lipid excretion from the digestive tract.

DLA-M, high fat feed (HFF) and HFF + DLA-M were added to the appropriate amount of distilled water to obtain smears and were later stained with Bodipy and Oil-Red-O. Polarized light microscopy revealed DLA-M crystals *in situ*. DLA-M crystals fixed lipids *in vitro*, as shown in Fig. 6f and Supplementary Fig. 1c. Because DLA-M does not enter the blood circulation^14^, we detected lipids that were immobilized by DLA-M in the gastrointestinal tract *in vivo*. Male CD-1 mice were fed a normal diet or a HFD for 3 days and were simultaneously treated with DLA-M (1 g/kg/day). As shown in Fig. 7a, we isolated the gastrointestinal tract (stomach, duodenum, jejunum, ileum and colon) to obtain frozen sections and content smears. Bodipy (Fig. 7b,c) and Oil-Red-O (Supplementary Fig.3a, b) staining indicated that DLA-M immobilized lipids in the gastrointestinal tract. The effect of DLA-M on preventing obesity development *via* immobilization of dietary lipids and enhancement of lipid excretion from the digestive tract is summarized in Fig. 8.

## Discussion

High-fat foods are rich in refined vegetable oils or fatty meats, and they are enjoyable in terms of taste. However, consumption of these foods results in lipids entering the blood stream from the digestive tract, which subsequently causes obesity. Traditional obesity prevention strategies are generally classified into four categories: reducing food intake, blocking fat absorption, increasing energy expenditure, and modulating lipid metabolism and storage^15^,^16^. Inhibition of fat absorption has largely been studied through faecal lipid analysis^17^,^18^,^19^,^20^, and whether DLA-M helps prevent obesity and its comorbidities has not been reported to date. Orlistat, a classical gastrointestinal fat blocker for obesity management, was approved by the FDA in 1999 as the first lipase inhibitor to block pancreatic lipase, thereby decreasing TG digestion^21^. By contrast, DLA-M, a dietary lipid-adsorbing agent, has the ability to adsorb and fix dietary lipids due to its lamellar structure and heterogeneous electric charge distribution; this ability allows it to enhance lipid excretion and thus, reduce lipid absorption by the digestive tract. Because this agent does not enter the circulatory system, it has no systemic side effects^14^. DLA-M may also be used as a safe and effective nutraceutical to manage obesity and its comorbidities.

DLA-M can prevent weight gain in rats fed a HFD (Fig. 1a,b). HFD + DLA-M-fed rats gained less weight than HFD-fed rats. In addition, food consumption did not differ between the three diet groups throughout the entire feeding period (Fig. 1c). The Lee obesity index, a predictive marker of percentage body fat in rats, dramatically decreased in the DLA-M treated group on the 15th day of treatment (Fig. 1d), indicating that the fat content of HFD + DLA-M-fed rats had decreased. Quantitative data indicated that the adipose tissues content decreased with DLA-M treatment; in particular, the Epi-WAT/BW ratio was 31.95% lower in the HFD + DLA-M-fed group than in the HFD-fed group. However, although the Per-WAT/BW and Mes-WAT/BW ratios decreased with DLA-M treatment (−21.25%, P = 0.11 and −25.62%, P = 0.07, respectively), the differences between the HFD + DLA-M-fed and HFD-fed groups were not significant (Fig. 2a,b). Adipocyte size was slightly affected in the early phase of weight gain; however, fat cell hypertrophy primarily occurred late in the process of fat deposition^22^. Interestingly, treatment with DLA-M inhibited adipocyte hypertrophy (Fig. 2c,d). Moreover, the adipocyte size distribution analysis showed that DLA-M-treatment specifically increased the number of small adipocytes, those with a size of 2000 to 6000 μm^2^, at the onset of HFD feeding (Fig. 2e). These results further suggest a novel role of DLA-M in adipocyte lipid accumulation in response to nutritional overload.

Hyperlipidaemia typically develops due to obesity and dietary choices^23^. Although hyperlipidaemia is asymptomatic, long-standing elevation of serum cholesterol can result in cardiovascular diseases^24^, including atherosclerosis (AS)^25^, coronary disease (CD)^26^ and hypertension^27^. Increasing TC and LDL-c levels and a decreasing HDL-c levels increase the risk of developing AS and CD^28^. Our data showed that in addition to preventing obesity, DLA-M clearly lowered TG, TC and LDL-c levels (Fig. 3a–c). Thus, we propose that DLA-M may have a hypolipidaemic effect, which would help preventing AS and CD, for those consuming a HFD. The liver is a vital internal organ and main organ regulator responsible for maintaining metabolic homeostasis using nutritional materials, such as sugars, lipids and proteins^29^. Oral administration of DLA-M dramatically decreased the HFD-induced increases in liver TG, TC and CE levels, and hepatic hypertrophy (Fig. 4). Our findings indicate that DLA-M inhibits hepatic lipid accumulation, which may play an important role in the prevention of the fatty liver associated with HFD consumption.

Bile acid plays an important role in regulating lipid metabolism in the intestine^30^. DLA-M had minimal adsorptive capacity for bile acid (Supplementary Fig. 2a, b). To mimic the physiological conditions in the intestinal environment in the presence and absence of bile, we treated both the 3T3-L1 mature adipocyte-conditioned medium, which contains large amounts of various lipids, and the intestinal contents of mice fed a normal diet or HFD with various concentrations of DLA-M. DLA-M adsorbed TGs and cholesterol in both systems (Fig. 6b–e).

The most interesting observation of our study was the immobilization of dietary lipids by DLA-M crystals *in vitro* and *in vivo*; this was directly observed *via* the combined use of polarized light microscopy and lipid staining with Bodipy and Oil-Red O. The ability of DLA-M to immobilize lipids results in the inhibition of lipid absorption by the gastrointestinal tract and promotion the amount of fecal lipid excretion with a high fat feeding (Fig. 7, Supplementary Fig. 3). Future studies are needed to make determine how DLA-M adsorbs lipids. Diosmectite, a DLA-M analogue, is an adsorbent that is widely used to treat of acute infectious diarrhoea in adults and children^31^. Constipation is a the significant side effect of diosmectite that occurs due to overdose^32^. To assess whether DLA-M causes constipation, we measured faecal water content. Our results indicated that DLA-M did not cause constipation in our experimental model. The positive and pleiotropic effects of DLA-M in terms of preventing obesity, hepatic steatosis and hyperlipidaemia suggest that it may be an excellent nutraceutical to adsorb excess lipids during the consumption of a fatty diet.

## Methods

### Materials and preparation of DLA-M

Mayer’s H&E and Oil Red-O were obtained from Sigma-Aldrich. BODIPY^®^493/503 was purchased from Thermo Fisher Scientific, Inc. DLA-M was isolated and purified from Xinjiang bentonite clay using the physical settlement method developed in our laboratory. First, bentonite clay was sufficiently ground and passed through a 180-mesh sieve. Second, distilled water was added to create a 5% dispersed suspension that was stirred for 25 to 30 min. The suspension was allowed to settle for 10 h, and the supernatant was filtered 3 times from centrifugation. Third, a suitable amount of flocculants was added to refine the seriflux, which was then concentrated, air dried and ground again. The final DLA-M product was an off-white powder, and it formed an insoluble gel when mixed with water.

### Animals and treatment

All experiments were carried out according to the guidelines and regulations of the Ethics and Animal Welfare Committee of Beijing Normal University. The methods were approved by the Ethics and Animal Welfare Committee, School of Life Science, Beijing Normal University (Approval No. CLS-EAW-2013-014). After one week of acclimatization, 2-month-old male SD rats (Vital River Laboratory Animal Technology Co. Ltd. Beijing, China), were randomly divided into three groups (n = 10, each) and fed with different diets. The experimental period was 25-days, one control group was fed a normal diet (chow); and another control group was fed a HFD (HFD: 77.75% normal diet, 21% fat and 1.25% cholesterol). And the treatment group was fed a HFD with DLA-M (HFD + DLA-M) at 1 g/kg BW by gavage daily at 5:00–6:00 p.m. Physiological saline was administered orally to the rats in the chow and HFD groups. Body weight, naso-Anal length and food intake were measured daily. Blood samples were collected at specific times *via* the caudal vein. The serum samples were stored at −80 °C until further analysis. At the end of the experimental period, the rats were starved for 12 h and then sacrificed. Faeces were collected during the final 5 days of the experimental period.

2-month-old male CD-1^®^ (ICR) mice were divided into three groups with the above-described treatments (n = 3–5, each). The HFD + DLA-M group received DLA-M (1 g/kg/day) for 3 days. The gastrointestinal system (stomach, duodenum, jejunum, ileum and colon) was freshly isolated to obtain frozen sections and content smears.

### Serum lipid profile

Blood samples were collected at specific times. TC, TG, HDL-c and LDL-c serum concentrations were determined using an Automatic Chemistry Analyzer (OLYMPUS AU400, Japan).

### Triglyceride, cholesterol and bile acid analysis

Liver and faecal lipid and bile acid concentrations were measured using the corresponding commercially available kits^33^,^34^. The tissue triglyceride assay kit (E1013), tissue total cholesterol assay kit (E1015) and tissue free cholesterol assay kit (E1016) were purchased from Applygen Technologies Co. Ltd. (Beijing, China). The total Bile acid Assay Kit (E003-1) was obtained from Nanjing Jiancheng Bioengineering Institute. (Nanjing, China). Faecal water content was measured using the Karl Fischer method^35^.

### DLA-M and lipid interaction analysis

For the *in vivo* experiment, the gastrointestinal system (stomach, duodenum, jejunum, ileum and colon) was freshly isolated. Then, intestinal segments rich in chyme were cut into 10 μm sections. Additionally, the intestinal contents of the mice were separated to make chyme smears that were stained with Bodipy or Oil Red-O. For the *in vitro* experiment, an appropriate amount of distilled water was added into DLA-M, HFF and HFF with DLA-M to obtain smears that were then stained with Bodipy or Oil-red O. Neutral lipids were visualized as green fluorescence at an absorbance wavelength of 488 nm using a fluorescence microscope (ZEISS Imager M1, Germany). Sections and smears were collected after lipid observation and were examined using a ZEISS Imager M1 polarized microscope to detect DLA-M crystals *in situ*^36^. 3T3-L1 mature adipocyte-conditioned medium was collected as previously^37^ and the content of the intestinal chime was separated as described above to mimic the physiological conditions of the intestinal environment for quantitative analysis.

### Adipose, liver and gastrointestinal tract histological analysis

Adipose and liver tissues were fixed in 4% formaldehyde, embedded in paraffin, cut into 5-μm sections, and stained with H&E following standard procedures^38^. Twenty microscopy images were obtained per treatment group, and the size of the adipocytes was analysed using Cell Profiler Software^39^,^40^. The gastrointestinal tract, liver and faecal frozen sections and relevant smears were visualized using Bodipy or Oil-Red O staining.

### Statistical analysis

Data obtained from three replicate experiments are expressed as the mean ± s.e.m. Differences between groups were statistically analysed using one-way ANOVA followed by Tukey’s multiple comparison tests as well as the unpaired t-test. Differences were considered to be statistically significant when the *P*- value was less than 0.05.

## Additional Information

**How to cite this article**: Xu, P. *et al.* Preventive obesity agent montmorillonite adsorbs dietary lipids and enhances lipid excretion from the digestive tract. *Sci. Rep.* **6**, 19659; doi: 10.1038/srep19659 (2016).

## Supplementary Material

Supplementary Information
