# Supplementary material for: Preventive obesity agent montmorillonite adsorbs dietary lipids and enhances lipid excretion from the digestive tract
Source: Sci Rep. 2016 Feb 19;6:19659. doi: 10.1038/srep19659 (PMC4759552; doi:10.1038/srep19659)
Supplement: Supplementary Information [file srep19659-s1.doc]

Preventive obesity agent montmorillonite adsorbs dietary lipids and enhances

lipid excretion from the digestive tract

Pengfei Xu^1^, Shu Dai^1^, Jing Wang^2^, Jun Zhang^2^, Jin Liu^1^, Fang Wang^3^ & Yonggong Zhai^1,2*^


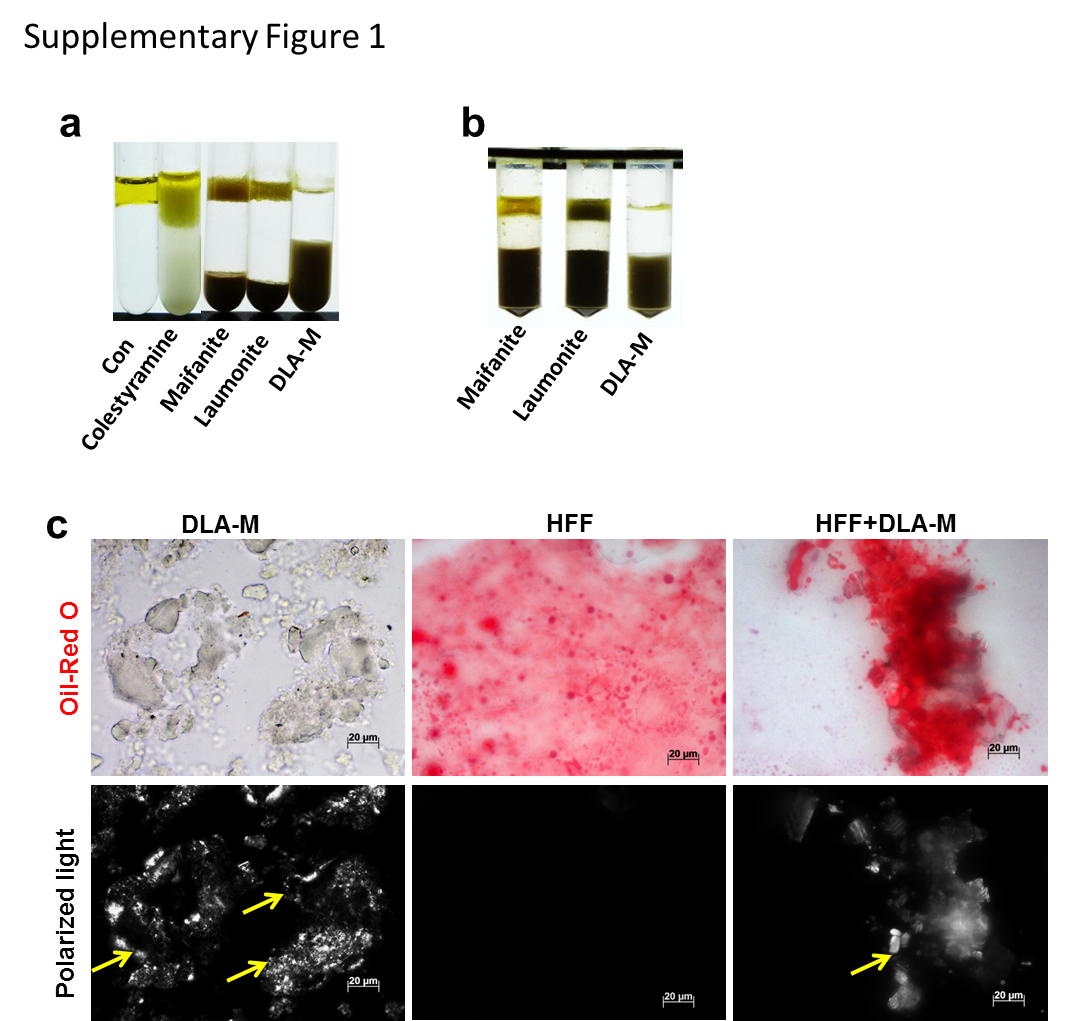


**Supplementary Figure 1 | DLA-M fixed diet lipid *in vitro*.** (a), DLA-M adsorbs vegetable oils: 800 μl normal saline and 200 μl siritch oil treated with 100mg/ml colestyramine or 400mg/ml maifanite, laumonite and DLA-M 400μl, respectively. (b), DLA-M adsorbs vegetable oils treated with maifanite, laumonite and DLA-M in a same volume. (c), Oil-Red O staining of DLA-M, HFF and HFF+DLA-M smears (neutral lipids appear red) and polarized light microscopy examination of DLA-M crystals (yellow arrows), scale: 20 μm. DLA-M, high-fat feed (HFF) and HFF+DLA-M (0.5 g HFF and 200 μl of DLA-M (400mg/ml) added to the appropriate amount of distilled water to obtain smears.


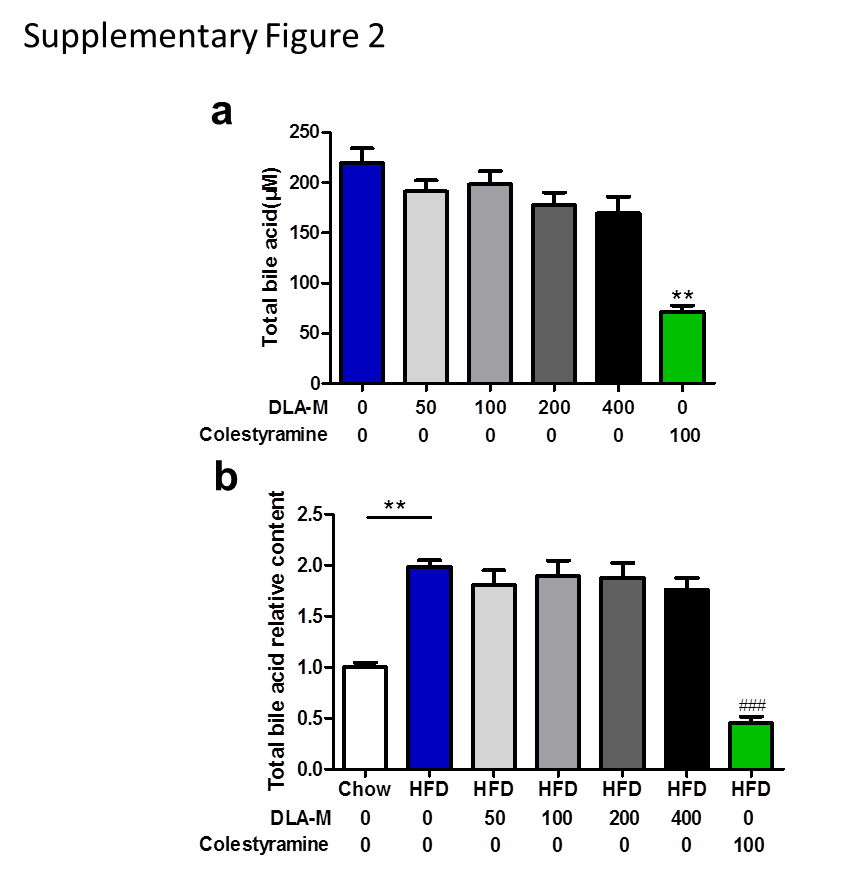


**Supplementary Figure 2 | DLA-M adsorbs little bile acid *in vitro.*** (a) Prepared 250μM cholic acid solution 500μl with alcohol, treated with 100μl DLA-M (0-400 mg/ml) or colestyramine 100mg/ml for 6 h, detected bile acid content in the supernatant. (b) Isolated intestinal contents of mice fed with normal diet or HFD: saline was added (1:20), the samples were treated with 0-400 mg/ml DLA-M or 100mg/ml colestyramine for 6 h, and then the relative contents of total bile acid was measured. Results are shown as the mean ± s.e.m. **, P < 0.01 compared with control, ###, P < 0.001 compared with HFD by one-way ANOVA with Tukey’s multiple comparison test.


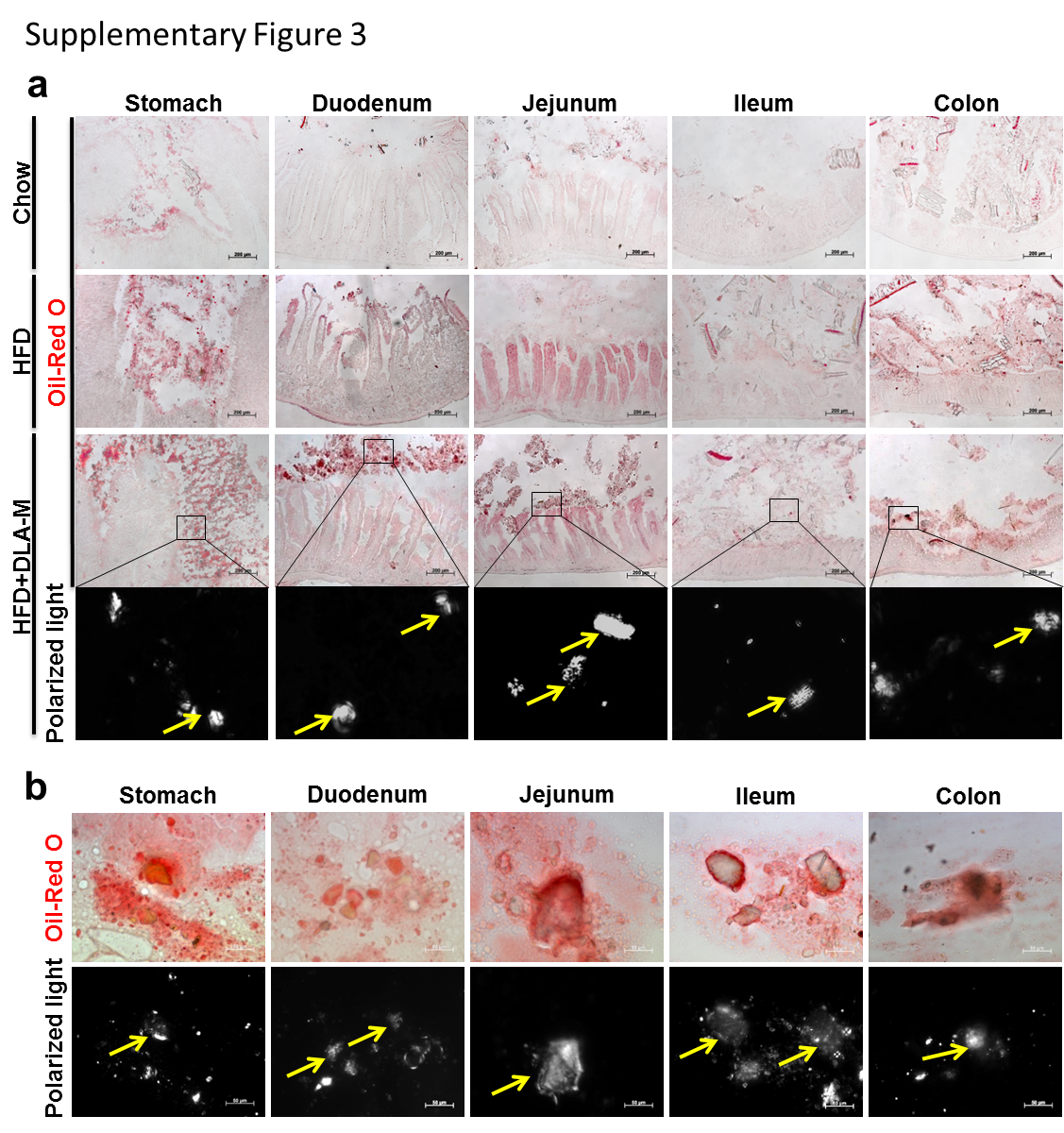


**Supplementary Figure 3 | DLA-M immobilized lipids in the gastrointestinal system *in vivo.***(a) Oil-Red O staining of digestive tract slices as described in mice fed a normal diet, HFD or HFD with DLA-M gavage (1 g/kg/day) for 3 days (scale: 200 μm). (b) Oil-Red O staining of gastrointestinal content smears of HFD+DLA-M mice; in the upper panels, neutral lipid appear red, and the lower panels polarized light microscopy examination images of DLA-M crystals (yellow arrows), scale: 50 μm.
